# Use of an anti-reflux catheter to improve tumor targeting for holmium-166 radioembolization—a prospective, within-patient randomized study

**DOI:** 10.1007/s00259-020-05079-0

**Published:** 2020-10-31

**Authors:** Caren van Roekel, Andor F. van den Hoven, Remco Bastiaannet, Rutger C. G. Bruijnen, Arthur J. A. T. Braat, Bart de Keizer, Marnix G. E. H. Lam, Maarten L. J. Smits

**Affiliations:** University Medical Center Utrecht, Utrecht University, Heidelberglaan 100, 3584 CX Utrecht, The Netherlands

**Keywords:** Radioembolization, Holmium-166, Colorectal cancer, Anti-reflux catheter, Surefire

## Abstract

**Purpose:**

The objective of this study was to investigate whether the use of an anti-reflux catheter improves tumor targeting for colorectal cancer patients with unresectable, chemorefractory liver metastases (mCRC) treated with holmium-166 (^166^Ho)-radioembolization.

**Materials and methods:**

In this perspective, within-patient randomized study, left and right hepatic perfusion territories were randomized between infusion with a Surefire® anti-reflux catheter or a standard microcatheter. The primary outcome was the difference in tumor to non-tumor (T/N) activity distribution. Secondary outcomes included the difference in infusion efficiency, absorbed doses, predictive value of ^166^Ho-scout, dose-response relation, and survival.

**Results:**

Twenty-one patients were treated in this study (the intended number of patients was 25). The median T/N activity concentration ratio with the use of the anti-reflux catheter was 3.2 (range 0.9–8.7) versus 3.6 (range 0.8–13.3) with a standard microcatheter. There was no difference in infusion efficiency (0.04% vs. 0.03% residual activity for the standard microcatheter and anti-reflux catheter, respectively) (95%CI − 0.05–0.03). No influence of the anti-reflux catheter on the dose-response rate was found. Median overall survival was 7.8 months (95%CI 6–13).

**Conclusion:**

Using a Surefire® anti-reflux catheter did not result in a higher T/N activity concentration ratio in mCRC patients treated with ^166^Ho-radioembolization, nor did it result in improved secondary outcomes measures.

**Trial registration:**

clinicaltrials.gov identifier: NCT02208804

**Supplementary Information:**

The online version contains supplementary material available at 10.1007/s00259-020-05079-0.

## Introduction

Radioembolization is an established treatment option for colorectal cancer patients with liver-dominant, chemorefractory, unresectable metastases (mCRCs) [1, 2].

Unfortunately, mCRC patients generally have relatively hypovascular, disseminated liver metastases, often leading to a suboptimal activity distribution [3, 4]. It has been hypothesized that the use of an anti-reflux catheter may improve treatment outcomes in two ways. First (partial), obstruction of the vascular lumen induces a decreased downstream pressure, possibly leading to better tumor targeting [5–10]. Also, the anti-reflux catheter causes a turbulent flow allowing particles to cross the laminar blood flow, leading to a more homogenous distribution [5]. In a small pilot study of nine patients with various tumor types, the use of an anti-reflux catheter led to a significant decrease in hepatic non-target embolization and a significant increase in activity deposition in the tumors [11].

Holmium-166 (^166^Ho)-microspheres (QuiremSpheres®, Quirem Medical, The Netherlands) were developed as an alternative to yttrium-90 (^90^Y)-microspheres. Instead of using ^99m^Tc-MAA as a predictor of activity distribution, ^166^Ho-scout (QuiremScout®, Quirem Medical, The Netherlands), a small batch of identical ^166^Ho-microspheres, can be used. This ^166^Ho-scout has proven to be a more accurate predictor of the distribution of the treatment dose [12]. ^166^Ho can be visualized in vivo by SPECT and MRI to assess activity distribution [13]. Precise quantification of ^166^Ho is possible using the Monte Carlo simulation that simultaneously compensates for scatter-, attenuation-, and collimator-detector response [14].

The aim of this study was to investigate whether the use of an anti-reflux catheter increases tumor targeting in comparison with a standard microcatheter in mCRC patients treated with ^166^Ho-radioembolization [15].

## Materials and methods

### Patients

The SIM study (“Surefire Infusion system® versus standard Microcatheter use during holmium-166 radioembolization”) was a single-center, within-patient, randomized controlled study (Clinicaltrials.gov: NCT02208804) (see also the Consort reporting checklist in the supplemental files). Patients with unresectable, chemorefractory, liver-dominant mCRC were eligible for this study if they had a pathologically confirmed diagnosis of CRC, hepatic metastases (≥ 1 cm and measurable on CT) in both the right and left hepatic arterial perfusion territory; a suitable arterial anatomy (not too tortuous vessels, with a large enough diameter to be accessible with the anti-reflux catheter), progressive disease after at least second-line systemic treatment, adequate liver-, renal-, and bone marrow function; and a life expectancy of > 3 months (see study protocol [15]). All patients provided written informed consent for participation in this study. The institutional review board provided ethical approval and the study was undertaken in accordance with the Declaration of Helsinki. An independent monitor verified all data.

### Procedures

Before treatment, patients underwent ^18^F-FDG PET/CT and a dual-phase contrast-enhanced CT. The hepatic arterial anatomy was assessed on the contrast-enhanced CT images and the perfusion territories of the left and right hepatic arteries (or their variants in case of aberrant vascular anatomy) were estimated. Metabolic hepatic tumor burden was assessed on the PET/CT images using ROVER software (ABX, Germany). Pre-treatment activity calculation was done using the standard formula for ^166^Ho-microspheres to reach an absorbed dose of 60 Gy in the target volume (in this study the whole liver) [16]:

$$ {IA}_{(MBq)}=\mathrm{liver}\ \mathrm{weight}\ \left(\mathrm{kg}\right)\ast 3780\ \left(\frac{MBq}{kg}\right) $$

**Fig. 1:**
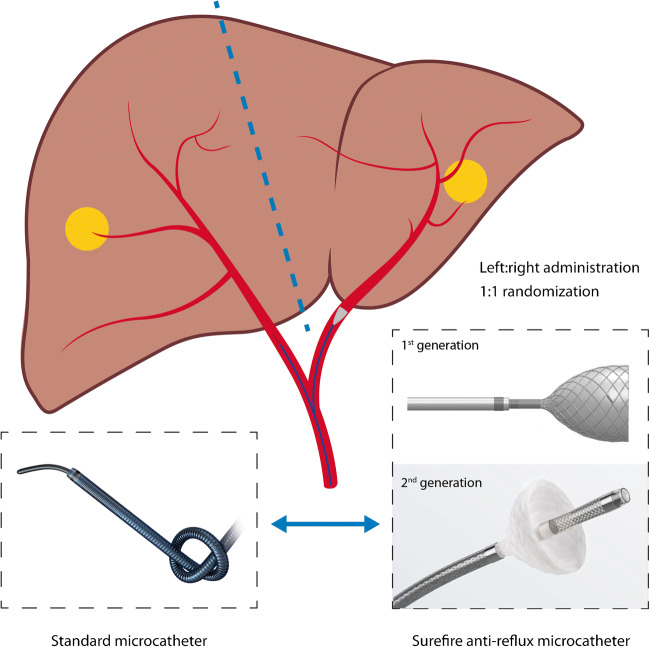
Schematic representation of within-patient randomized treatment with a standard microcatheter in the right hepatic artery and an anti-reflux catheter in the left hepatic artery. First-generation anti-reflux systems were used until August 2017 and were then replaced by the second-generation anti-reflux systems

In which IA is the injected activity and 3780 is the constant specific for ^166^Ho. The prescribed activity was split according to the perfusion volume of the left and right hepatic arteries as estimated on pre-treatment contrast-enhanced CT. Before treatment, patients’ perfusion territories were randomized by the investigator between injection with a standard microcatheter and an anti-reflux catheter, using a computer-generated stratified block randomization with the difference in tumor burden (above or below 10%) as a stratification factor. The result of randomization was applied for both the ^166^Ho-scout and the therapeutic activity (Fig. 1). On the day of treatment, randomization was disclosed to the interventional radiologist. Two types of anti-reflux catheters were used during the study period. The first version of the Surefire® anti-reflux catheter (TriSalus Life Sciences, Westminster, CO, USA) was initially used, but this catheter became unavailable during the course of the study. Since January 2018, the updated Surefire® Precision infusion system was used. The standard microcatheter was a Progreat 2.4-F or 2.7-F microcatheter (Terumo Europe NV, Leuven, Belgium). The price of the anti-reflux catheter was €1500 excluding VAT and the prices of the standard microcatheters were €275–€295 excluding VAT. Patients received ^166^Ho-scout in the morning, followed by ^166^Ho-SPECT/CT imaging. In the absence of relevant extrahepatic deposition, patients received the therapeutic activity in the afternoon as part of a 1-day protocol. Three to 5 days later, another ^166^Ho-SPECT/CT was acquired to assess the therapeutic absorbed dose distribution. SPECT-imaging cannot be performed directly after administration, since the abundance of gamma photons invokes detector dead-time: the recorded photon produces a pulse of a certain duration during which no second pulse can be detected [14]. The distribution on the post-treatment ^166^Ho-SPECT/CT was the basis for the primary endpoint.

SPECT/CT imaging after ^166^Ho-scout and after ^166^Ho-treatment was performed on a Symbia T16 system (Siemens Health Care) with a medium-energy collimator. Images were acquired on a 128 *128 matrix 120 angles over a 360° non-circular orbit (30 s/projection) with an energy window of 81 keV. Afterward, a low-dose CT scan was fused with the SPECT images. The reconstruction of the data was done using the Utrecht Monte Carlo System software [17].

**Fig. 2:**
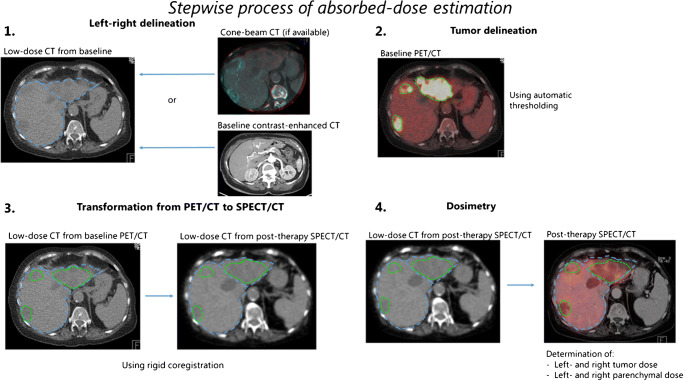
Stepwise process of absorbed-dose estimation after treatment. First, left- and right perfusion territories were manually delineated on the low-dose CT from the baseline [^18^F]-FDG PET/CT, based on the cone-beam CTs (if available) or the baseline contrast-enhanced CTs. Afterward, tumors were automatically defined on the baseline [^18^F]-FDG PET/CT using a threshold-based approach. Then, the low-dose CTs of the baseline [^18^F]-FDG PET/CT and the post-treatment ^166^Ho-SPECT/CT were coregistered. Using a rigid transformation, the volumes of interest of the tumors and the healthy liver tissue (the left and right perfusion territories) were transferred to the ^166^Ho-SPECT/CT and absorbed doses were obtained

After treatment, toxicity was assessed during a telephone consultation at 2 weeks after treatment and by physical and laboratory examination at 1 and 3 months after treatment. Adverse events were graded according to the Common Terminology Criteria for Adverse Events (CTCAE) version 5.0. The maximum severity of each adverse event was reported. Response to treatment was assessed on PET/CT and contrast-enhanced CT 3 months after treatment, blinded for catheter allocation. Response analyses were based on the metabolic response to treatment, based on a change in total lesion glycolysis between baseline and 3 months post-treatment, according to the PERCIST guidelines [18]. The primary outcome of this study was the difference in tumor to non-tumor (T/N) activity concentration ratio between the right and left liver lobes, randomized between administration with an anti-reflux and a standard microcatheter. Secondary outcomes included the difference in infusion efficiency (the percentage of activity administered), absorbed doses, the predictive value of the ^166^Ho-scout, the dose-response relation, and survival. For the analyses, the contours of the tumors and the parenchyma were used that were identified on the baseline [^18^F]-FDG PET/CT. The left/right lobe delineation was done on the accompanying low-dose CTs of the baseline [^18^F]-FDG PET/CT using the cone-beam CT images on the side as a reference. The tumor contours were obtained using a threshold-based approach, based on the PERCIST guidelines. The resulting volumes of interest were transferred from the [^18^F]-FDG PET/CT to the ^166^Ho-SPECT/CT using a rigid coregistration of the accompanying low-dose CTs, as described before [19] (Fig. 2).

### Statistical analyses

The sample size calculation, based on a difference of 0.4 in mean tumor to non-tumor (T/N) activity ratio between the catheters, showed that at least 23 patients needed to be treated [15]. The intent was to treat 25 patients [15]. The differences in mean post-treatment T/N activity ratio and mean tumor- and healthy liver-absorbed dose on SPECT/CT between administration with an anti-reflux catheter and a standard microcatheter were assessed using a paired *t* test. The infusion efficiency was calculated as the percentage residual activity and compared using a McNemar’s test for paired data. The predictive value of the ^166^Ho-scout was assessed using the Bland–Altman analysis. The relation between tumor-absorbed dose and response was best explained using a linear mixed-effects regression model, using a random intercept per patient, to account for correlation of tumors within patients. The influence of the anti-reflux catheter on tumor response was analyzed with logistic regression. Analyses were primarily performed according to the intention-to-treat (ITT) principle. Per-protocol analyses were also performed. A subgroup analysis was performed in patients in whom the anti-reflux catheter was deployed in the right hepatic artery, under the hypothesis that its effect on hemodynamics and dose distribution would be most notable in wide vessels. Furthermore, a subgroup analysis was performed in liver lobes treated with the anti-reflux catheter only, to evaluate the influence of spasm (as evident during angiography) on T/N activity concentration ratio. Overall survival was defined as the interval between treatment and death from any cause. Cox regression models were made using Firth’s correction for small sample bias [20]. Analyses were performed using the R statistical software for Windows, version 3.6.2. We report effect estimates with associated 95% confidence intervals and corresponding two-sided *p* values.

## Results

**Fig. 3:**
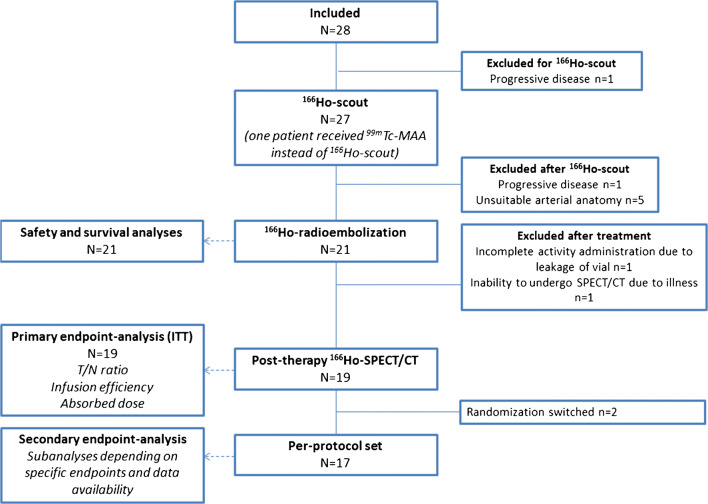
Flowchart of study procedures in included patients

This study was discontinued prematurely because of slow recruitment and a high drop-out rate. In total, 28 patients were included in this study between June 2014 and April 2019. Two patients were diagnosed with rapidly progressive disease and no longer meeting the inclusion criteria and excluded before administration of ^166^Ho-scout and/or ^166^Ho-radioembolization. In five patients (18%), an anti-reflux catheter could not be used because of unsuitable vascularity, meaning that because of vessel size, tortuosity, or the occurrence of vasospasm, an adequate injection position with the anti-reflux catheter could not be obtained. Twenty-one patients received ^166^Ho-radioembolization using the anti-reflux catheter (Table 1, Fig. 3). Median time from pre-treatment imaging using [^18^F]-FDG PET/CT to treatment was 14 days (range 6–42 days) and the median time from pre-treatment imaging to post-treatment ^166^Ho-SPECT/CT was 17 days (range 9–46 days). Administration characteristics are listed in Table 2. In two of these patients, catheter allocation was switched during treatment because of vessel size and tortuosity. In one patient, due to a vial deficiency, only a small part (15% in one lobe) of the activity was injected into the liver. Furthermore, follow-up imaging was not (fully) available in two patients and a post-therapy ^166^Ho-SPECT/CT was not acquired in one patient (Fig. 1). Sixteen patients were treated with the first version of the Surefire® anti-reflux catheter and five patients were treated with the updated second version: the Surefire Precision infusion system®. In six of 21 treated patients (29%), vasospasm occurred during the use of the anti-reflux catheter, both with the initial version (5/16 patients) and with the newer Surefire Precision system® (1/5 patients). Nitroglycerin was administered in 18/21 Surefire® injections during vasospasm or as prophylaxis to prevent vasospasm. Adverse device effects are listed in Table 3.

**Table 1:** Patient and treatment characteristics

Characteristic	*n* or median + range	
	All included patients(*n* = 28)	Treated population (*n* = 21)
Gender		
Male	17	13
Female	11	8
Age (years)	60 (37–83)	63 (45–83)
WHO performance score		
0	18	16
1	9	5
2	1	0
Primary tumor location		
Left	21	14
Right	7	7
Previous therapy		
Locoregional (liver)	3	3
Metastasectomy	3	3
Systemic	28	21
5-FU	9	6
Bevacizumab	24	18
Capecitabine	24	19
Cetuximab	2	2
Folinic acid	9	6
Irinotecan	19	14
Oxaliplatin	26	19
Panitumumab	9	7
Regorafenib	1	1
TAS-102	3	1
Trifluridine + tipiracil	1	0
Extrahepatic disease before treatment		
Lymph node	12	9
Lung	9	7
Ovaries	1	0
Peritoneum	1	0
No	11	9
Liver volume (mL)	1968 (1560–3134)	1923 (1428–2952)
Metabolic tumor volume (mL)	271 (88–769)	311 (70–769)
Tumor load (%)	15 (5–35)	16 (5–26)
Total prescribed activity (MBq)	7607 (4850–12,782)	7862 (4325–12,782)
Total residual activity (MBq)	346 (98–4107)	495 (98–4107)
Administered therapeutic activity (MBq)	7119 (3142–12,386)	7099 (3142–12,386)
Administered ^166^Ho scout activity (MBq)	246 (163–156)	238 (163–356)

**Table 2:** Administration characteristics of 21 treated patients

Characteristic	*n* or median + range			
	Standard microcatheter	Anti-reflux catheter	Anti-reflux catheter–Surefire Infusion System (1st generation)	Anti-reflux catheter–Surefire Precision Infusion System (2nd generation)
Perfusion territory volume (mL)	711 (157–1901)	1104 (462–1685)	938 (462–1685)	711 (704–1271)
Tumor volume	101 (14–417)	175 (43–379)	175 (43–379)	178 (86–256)
Tumor burden (%)	15 (2–46)	16 (6–72)	17 (6–72)	13 (8–20)
Anatomy				
Standard	13	16	12	4
Replaced main perfusion territory artery	6	4	3	1
Early branching pattern	2	1	1	0
Coil-embolization*	1	0	0	0
Total administered activity (MBq)	2206 (671–5867)	3525 (680–5995)	4443 (1777–5525)	4075 (680–5995)

*Coil-embolization of a main perfusion territory artery

**Table 3:** Adverse device effects in 21 included patients

	Standard microcatheter	Anti-reflux catheter	Anti-reflux catheter–Surefire Infusion System (1st generation)	Anti-reflux catheter–Surefire Precision Infusion System (2nd generation)
Spasm	1/21	5/21	5/16	1/5
Stasis	3/21	3/21		
Unstable injection position	0/21	3/21	3/16	0/5
Inability to reach the desired injection position	0/21	5/21 (LHA *n* = 4)	5/16	0/5
Inadvertent vessel occlusion	0/21	1/21	0/16	1/5

### Treatment

**Fig. 4:**
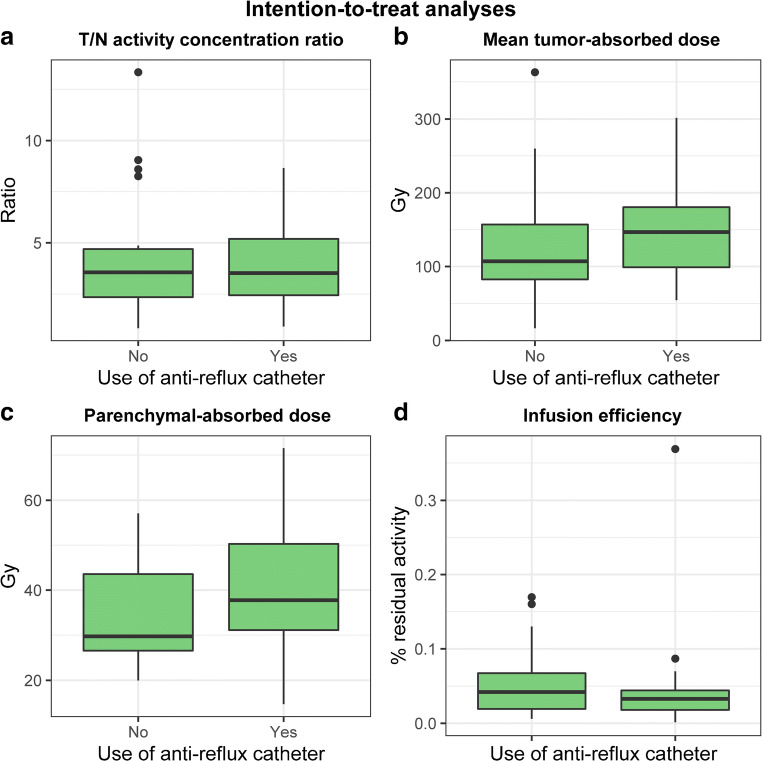
**a**–**d** Intention-to-treat analyses of the effect of anti-reflux catheter on T/N activity concentration ratio (**a**), mean tumor-absorbed dose (**b**), mean parenchymal-absorbed dose (**c**), and infusion efficiency (**d**)

The ITT analyses were performed on 19/21 evaluable patients, including the two patients in whom catheter allocation was switched (i.e., the volume that was intended to be treated with the anti-reflux catheter was counted as volume treated with the anti-reflux catheter and vice versa). In one patient, ^166^Ho-scout was not available due to a production failure, and treatment simulation was performed using ^99m^Tc-MAA, but this patient was still evaluable for the primary endpoint. The patient with incomplete activity administration due to vial leakage (in one lobe, only 15% of calculated activity was administered) and the patient without a post-therapy ^166^Ho-SPECT/CT were excluded from these analyses. The median T/N activity concentration ratio with the use of the anti-reflux catheter was 3.2 (range 0.9–8.7) versus 3.6 with a standard microcatheter (range 0.8–13.3) (difference in median − 0.4, 95%CI − 1.22–1.29, *p* = 0.92) (Fig. 4). The median T/N activity concentration ratio with the anti-reflux catheter in the presence of spasm was 3.5 (range 2.4–4.7) versus 3.7 (range 0.9–8.7) without the occurrence of spasm (*p* = 0.31, 95%CI −3.95–1.55). Both the median tumor-absorbed dose and the parenchymal-absorbed dose were (not-significantly) higher with the use of the anti-reflux catheter (difference in median tumor-absorbed dose + 25 Gy, 95%CI − 27–62, *p* = 0.54 and difference in median parenchymal-absorbed dose + 8 Gy, 95%CI − 0.2–15.2, *p* = 0.06) (Fig. 4b,c). There was no difference in infusion efficiency between the use of the anti-reflux catheter (median residual activity 0.03%, range 0.001–0.37) and the standard microcatheter (median residual activity 0.04%, range 0.006–0.17) (difference in median − 0.01%, 95%CI − 0.05–0.03, *p* = 0.93) (Fig. 4d).

The per-protocol analyses were performed in 17 patients. Median T/N activity concentration ratio with the anti-reflux catheter was 3.2 (range 0.9–8.7); with the standard microcatheter 3.6 (range 0.8–13.3) (*p* = 0.82, 95%CI − 1.19–1.24). Median tumor-absorbed dose was 129 Gy (range 55–302) with the anti-reflux catheter versus 107 Gy (range 17–363) with the standard microcatheter (*p* = 0.61, 95%CI − 33–49). Median parenchymal-absorbed dose was 38 Gy (range 15–67) with the anti-reflux catheter and 30 Gy (range 20–57) with the standard microcatheter (*p* = 0.13, 95%CI − 3–14). Infusion efficiency with the anti-reflux catheter was 0.03 (range 0.0012–0.37) versus 0.04 (range 0.006–0.17) with the standard microcatheter (*p* = 0.53, 95%CI − 0.06–0.17) (Figure S1a–d).

**Fig. 5:**
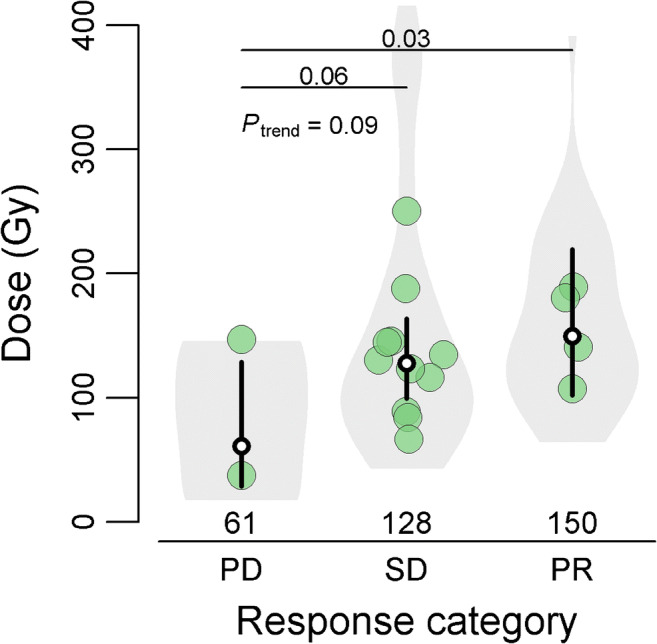
Relationship between mean tumor-absorbed dose per patient and metabolic response to treatment at a three-month follow-up. The bullets show the mean tumor-absorbed dose per patient. Black vertical lines are the 95%CIs of the mean doses per response category, with the white dot in the middle indicating the mean tumor-absorbed dose per response category. This figure is based on the linear mixed-effects regression model as described in Table 3

At a tumor-level, a significant dose-response relationship was established. The mean tumor-absorbed dose in tumors with complete metabolic response was on average 138% higher than in progressive tumors (222 Gy vs. 103Gy, respectively; 95%CI 8–243%). The mean tumor-absorbed dose was 3.8% higher with the use of the anti-reflux catheter than with the standard microcatheter (170 Gy vs. 145 Gy, respectively; 95%CI − 37–71%, *p* = 0.89). The odds ratio for metabolic response (complete or partial response) with the use of the anti-reflux catheter was 0.75 (95%CI 0.25–2.25). Tumor- and patient-level metabolic response is summarized in Table S1 and Fig. 5.

### Exploratory sub-analysis of the impact of anti-reflux catheter on the right hepatic artery only

The anti-reflux catheter was deployed in the right hepatic artery in twelve patients (per protocol analysis). The median T/N activity concentration ratio with the anti-reflux catheter was 4.0 (range 0.9–8.7) versus 3.8 with the standard microcatheter (range 0.8–9.0) (difference in median + 0.2, 95%CI − 1.11–2.32, *p* = 0.42).

### Safety

Grade ≥ 3 laboratory toxicity was present in three patients (14%), and four patients (19%) experienced grade ≥ 3 clinical toxicity. Two deaths occurred within 3 months after treatment: one patient died of disease progression (5%), the other of radioembolization-induced liver disease (5%) (Table S2. Median overall survival was 7.8 months (95%CI 6.4–12.9).

### Agreement between ^166^Ho-scout and ^166^Ho-therapy

The agreement between the dose distribution on ^166^Ho-scout and ^166^Ho-therapy was calculated for 17 patients. Four patients were excluded from the analysis, for the following reasons: treatment simulation by ^99m^Tc-MAA (*n* = 1; ^166^Ho-scout was not available due to a production failure and treatment simulation was performed using ^99m^Tc-MAA), no post-treatment ^166^Ho-SPECT/CT performed (*n* = 1), stasis during treatment (defined as retrograde flow into adjacent arteries) (*n* = 1), and ^166^Ho-scout activity for the left hepatic territory abusively administered in the right hepatic artery (*n* = 1). Using the anti-reflux catheter led to a substantially worse agreement for estimating the tumor-absorbed dose with ^166^Ho-scout (95% limits of agreement − 58 Gy and + 49 Gy) than when using the standard microcatheter (95% limits of agreement − 27 Gy and + 29 Gy) (Figure S2a, b). The agreement for estimating the parenchymal dose was similar between both catheter types (95% limits of agreement − 3 Gy and + 2 Gy for the anti-reflux catheter and − 3 Gy and + 3 Gy for the standard microcatheter) (Figure S2c, d).

## Discussion

Colorectal cancer liver metastases are challenging to treat with radioembolization. These tumors are often diffusely metastasized throughout the liver and are hypovascular compared to other tumor types [3, 4]. As a consequence, tumor-targeting is often poor and response rates after radioembolization in mCRC patients are modest [21, 22]. The hypothesis was tested that the use of an anti-reflux catheter improves tumor targeting during radioembolization. However, in this within-patient RCT, the use of the anti-reflux catheter did not lead to significant differences in T/N activity concentration ratio, tumor- and parenchymal-absorbed dose, or infusion efficiency.

To our knowledge, this is the first prospective study in humans investigating the supposed improved tumor targeting when using the Surefire® anti-reflux catheter for radioembolization. In the first study investigating this anti-reflux catheter, renal artery embolization with tantalum beads in a porcine model was performed with a standard microcatheter (*n* = 3) versus an anti-reflux catheter (*n* = 3). Embolization efficiency was 99.9% ± 1 with the anti-reflux catheter, versus 72% ± 13 with a standard microcatheter [9]. Early studies found that infusion efficiency with the anti-reflux catheter was significantly improved due to a decrease in blood pressure in the downstream vascular territory [7, 8]. Mean blood pressure with the tip closed was 79 mmHg versus 58 mmHg with the tip expanded [7]. Besides a higher infusion efficiency, the use of anti-reflux catheters was found to lead to a higher tumor-absorbed dose in a mixed tumor-type cohort of nine patients who received pre-treatment infusion with ^99m^Tc-MAA twice, using both the anti-reflux catheter and a standard microcatheter. A relative increase in tumor deposition ranged from 33 to 90% [11]. Most studies were performed with the first version of the Surefire® anti-reflux catheter. A new version, the Surefire Precision Infusion System®, was introduced in January 2018 and is expected to have similar effects, although it has a different deployment mechanism: the anti-reflux umbrella is no longer situated at the tip of the catheter but is positioned slightly more proximal. Also, the catheter-shaft of the later version is less rigid. In contrast to the studies described before, we did not find significant differences between the anti-reflux catheter and the standard microcatheter. Possible reasons for this are the differences in patient population (only mCRC versus mixed tumor-type cohorts/even porcine models), embolic device (^166^Ho versus ^90^Y, ^99m^Tc-MAA, tantalum beads, or chemoembolization particles), and treatment approach (lobar versus segmental). In addition, the manufacturer of the anti-reflux was in no way involved in this investigator-initiated study.

We met several challenges while conducting this innovatively designed trial. Ultimately, this study was stopped prematurely due to slow accrual and a high drop-out rate. During weekly tumor boards, possible candidates were screened for eligibility. Based on contrast-enhanced CT, many patients were already deemed unsuitable because of their vascular anatomy (mostly because of arteries that were deemed too small or too tortuous for the relatively rigid anti-reflux catheter). Nevertheless, despite careful pre-selection and studying anatomy before treatment, five included patients (18%) were still excluded during angiography because the desired injection position could not be obtained with the anti-reflux catheter. Positioning was challenging as the catheter sometimes moved forward with the deployment of the anti-reflux system, rendering it difficult to reach a stable injection position. Furthermore, with the use of the anti-reflux catheter, vasospasm occurred very frequently (in 24% of cases), which required the administration of intra-arterial nitroglycerin in most cases. The effect of nitroglycerin on the T/N ratio is unknown. Vasospasms occurred probably because of the relatively rigid catheter shaft and due to contact between the deployed anti-reflux system and the vessel wall. These technical difficulties were most pronounced with the first version of the anti-reflux catheter, as the shaft of the second-generation catheter was more flexible and the anti-reflux system could be more easily deployed while maintaining a stable injection position.

The strengths of this study were the within-patient randomized study design and the homogenous patient population. The main limitation of this study was the small number of patients, which may have caused potential differences in primary or secondary outcomes to remain undetected. However, in our study, no effect (even a small negative effect) of the anti-reflux catheter on the primary and secondary outcomes was found. Based on our results, it is unlikely that with enough power, a large positive effect of the anti-reflux catheter will be seen. Also, the frequent occurrence of technical adverse events with the anti-reflux catheter likely contributed to the lack of a positive influence on treatment outcomes. The occurrence of vasospasm, for example, probably had an influence on activity distribution. Another limitation is the time between pre-treatment imaging with [^18^F]-FDG PET/CT and post-treatment ^166^Ho-SPECT/CT. Although much effort was done to limit the time between baseline imaging and treatment, an increase of tumor and/or hepatic volume may have occurred, leading to imperfections in segmentation. Furthermore, in this study, the perfusion volumes of the left and right hepatic arteries were estimated on pre-treatment CT. The more accurate method of using perprocedural C-arm CT with contrast injection via a microcatheter in the left and right hepatic arteries was logistically not possible since patients underwent the work-up angiography on the same day as the treatment angiography and ^166^Ho-microspheres need to be ordered 7 days in advance.

This study had a within-subject design, which has several advantages. First, patients serve as their own control, limiting possible confounding by extraneous patient variables [23] and requiring fewer subjects to detect meaningful effects. However, a within-patient design is only applicable, when the treatment of one body part (in our case functional liver half) is unlikely to affect the other body part for the outcome under study. While designing this study, we judged that the technical nature of the relationship between catheter design and particle distribution was suitable for this study design, because we assumed that this interplay is limited to local fluid-dynamics and that systemic carry-across effects are unlikely [24]. If, however, systemic effects (e.g., the activation of vasogenic factors during the occurrence of near-stasis) do play a role, they may have negated potential differences in preferential tumor-targeting between the anti-reflux and standard microcatheter. In our patient population, some tumors were located close to the so-called watershed areas and may actually have received blood supply from both perfusion territories (although this was not observed on cone-beam CT). Another disadvantage of our design was that although patient-level characteristics are accounted for, there are still within-patient characteristics that may cause a random error. The anti-reflux catheter was, for example, much easier deployed in the right hepatic artery, as this often was a much larger, less tortuous vessel. The new version of the anti-reflux catheter was (due to randomization, not deliberately) only used in the right hepatic arteries, which may explain the difference in the occurrence of vasospasm between the two anti-reflux catheter versions. In our experience, the standard microcatheter used in this study had a much more flexible shaft and was therefore superior in tracking the guidewire and navigating the liver vasculature, when compared to both versions of the anti-reflux catheter. Also, although accounted for in the randomization, the tumor burden was not always equal between perfusion territories.

The agreement between the ^166^Ho-scout and ^166^Ho-therapy dose distribution in our study was high and in line with a previous study [12]. These results support the use of ^166^Ho-scout for treatment planning. Surprisingly, the agreement with the anti-reflux catheter at a tumor level was worse compared with a standard microcatheter. The mechanical pressure of the anti-reflux catheter on the vascular wall may have caused a larger variation in flow between the administration of ^166^Ho-scout and ^166^Ho-therapy.

## Conclusion

In this study, no differences in post-treatment T/N activity concentration ratio, tumor- and parenchymal-absorbed dose, and infusion efficiency were found between the use of an anti-reflux catheter and a standard microcatheter in mCRC patients treated with ^166^Ho-radioembolization.

## Electronic supplementary material

ESM 1 Figure S1a-d. Per protocol analysis of effect of anti-reflux catheter on T/N activity concentration ratio (a), mean tumor-absorbed dose (b), mean parenchymal-absorbed dose (c) and infusion efficiency (d). (JPG 1654 kb)

ESM 2 Figure S2a-d. Bland-Altman plot for the agreement between estimated absorbed doses based on ^166^Ho-scout and the actual absorbed doses with ^166^Ho-therapy. Tumor-absorbed doses with the anti-reflux catheter and the standard microcatheter are visualized in A,B. Figs. C and D show the estimated parenchymal-absorbed doses for the two catheter types. (PNG 2539 kb)

High resolution image (TIF 11041 kb)

ESM 3 (DOCX 13 kb)

ESM 4 (DOCX 17 kb)

ESM 5 (DOCX 55 kb)
