# Supplementary figures and images for: Use of an anti-reflux catheter to improve tumor targeting for holmium-166 radioembolization—a prospective, within-patient randomized study
Source: Eur J Nucl Med Mol Imaging. 2020 Oct 31;48(5):1658–68. doi: 10.1007/s00259-020-05079-0 (PMC8113291; doi:10.1007/s00259-020-05079-0)

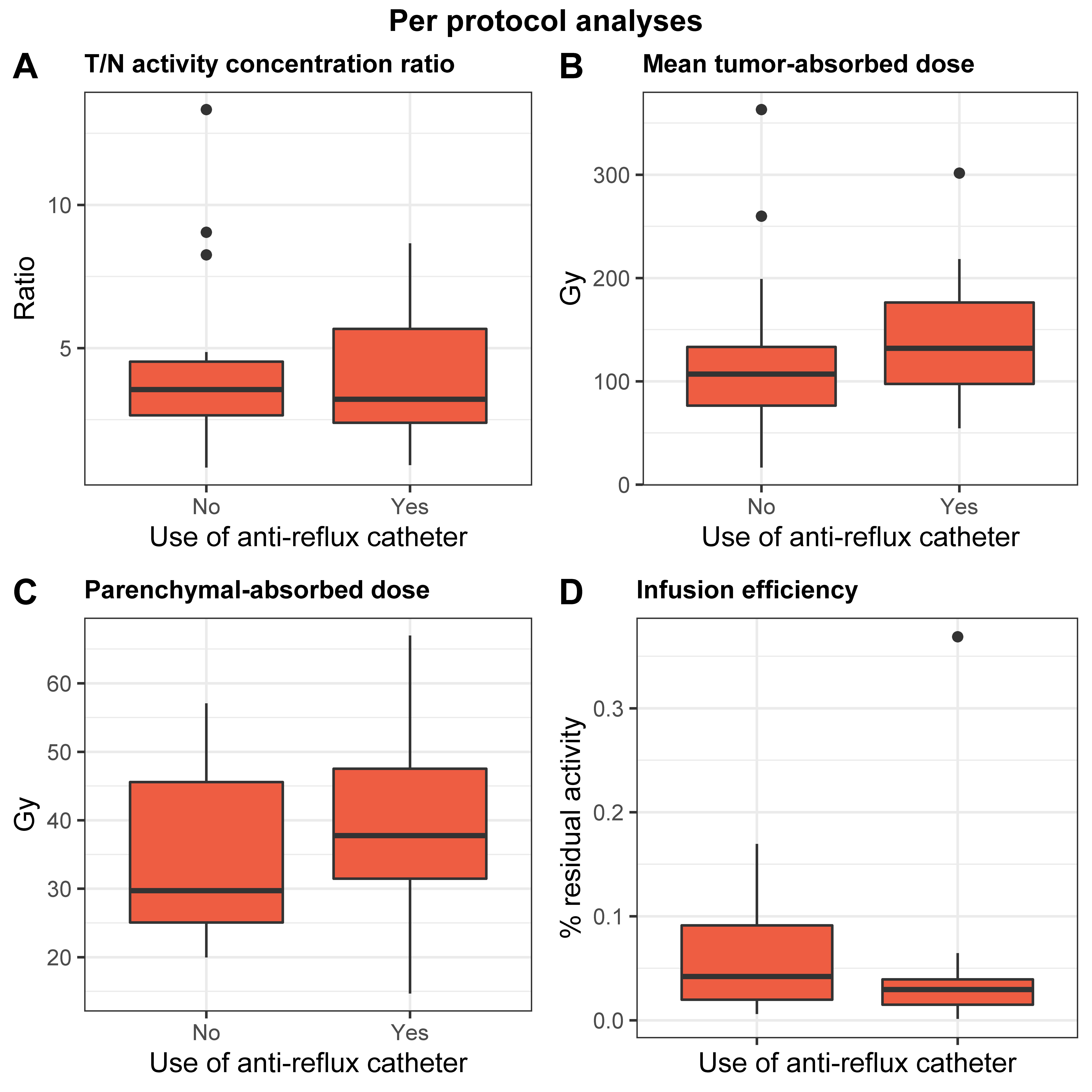

Supplement: Supplementary file 1 — Figure S1a-d. Per protocol analysis of effect of anti-reflux catheter on T/N activity concentration ratio (a), mean tumor-absorbed dose (b), mean parenchymal-absorbed dose (c) and infusion efficiency (d). (JPG 1654 kb) [file 259_2020_5079_MOESM1_ESM.jpg]

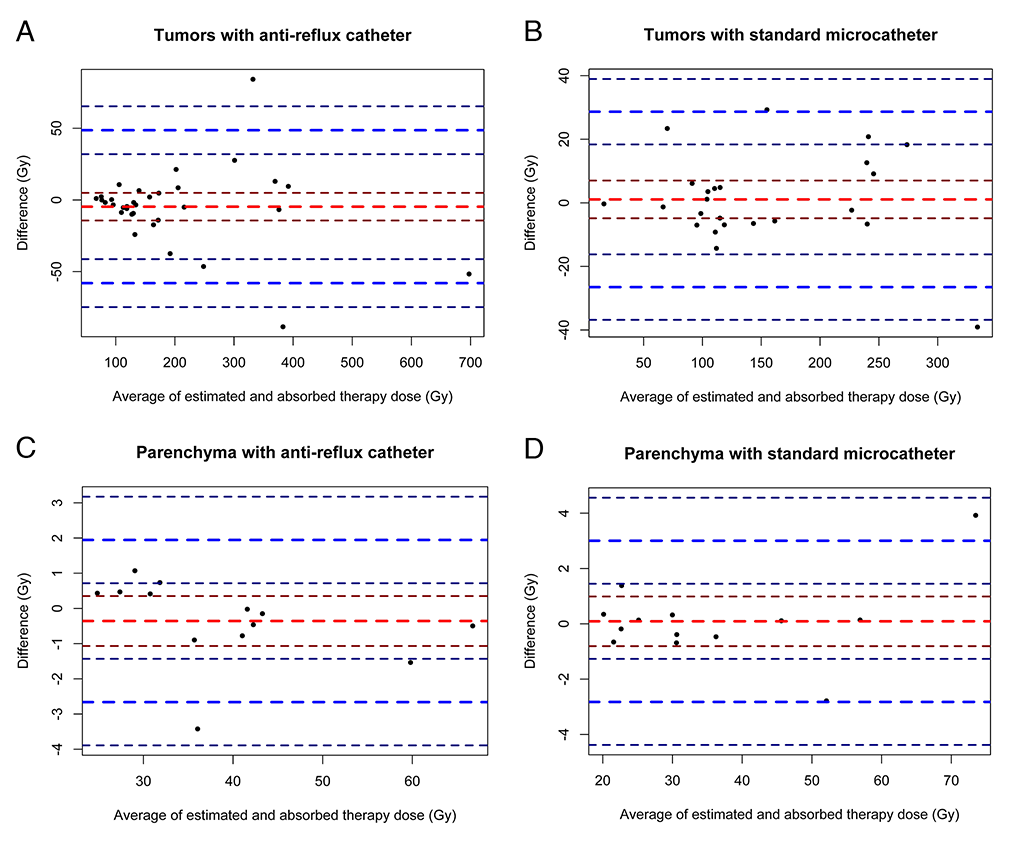

Supplement: Supplementary file 2 — Figure S2a-d. Bland-Altman plot for the agreement between estimated absorbed doses based on 166Ho-scout and the actual absorbed doses with 166Ho-therapy. Tumor-absorbed doses with the anti-reflux catheter and the standard microcatheter are visualized in A,B. Figs. C and D show the estimated parenchymal-absorbed doses for the two catheter types. (PNG 2539 kb) [file 259_2020_5079_Fig6_ESM.png]

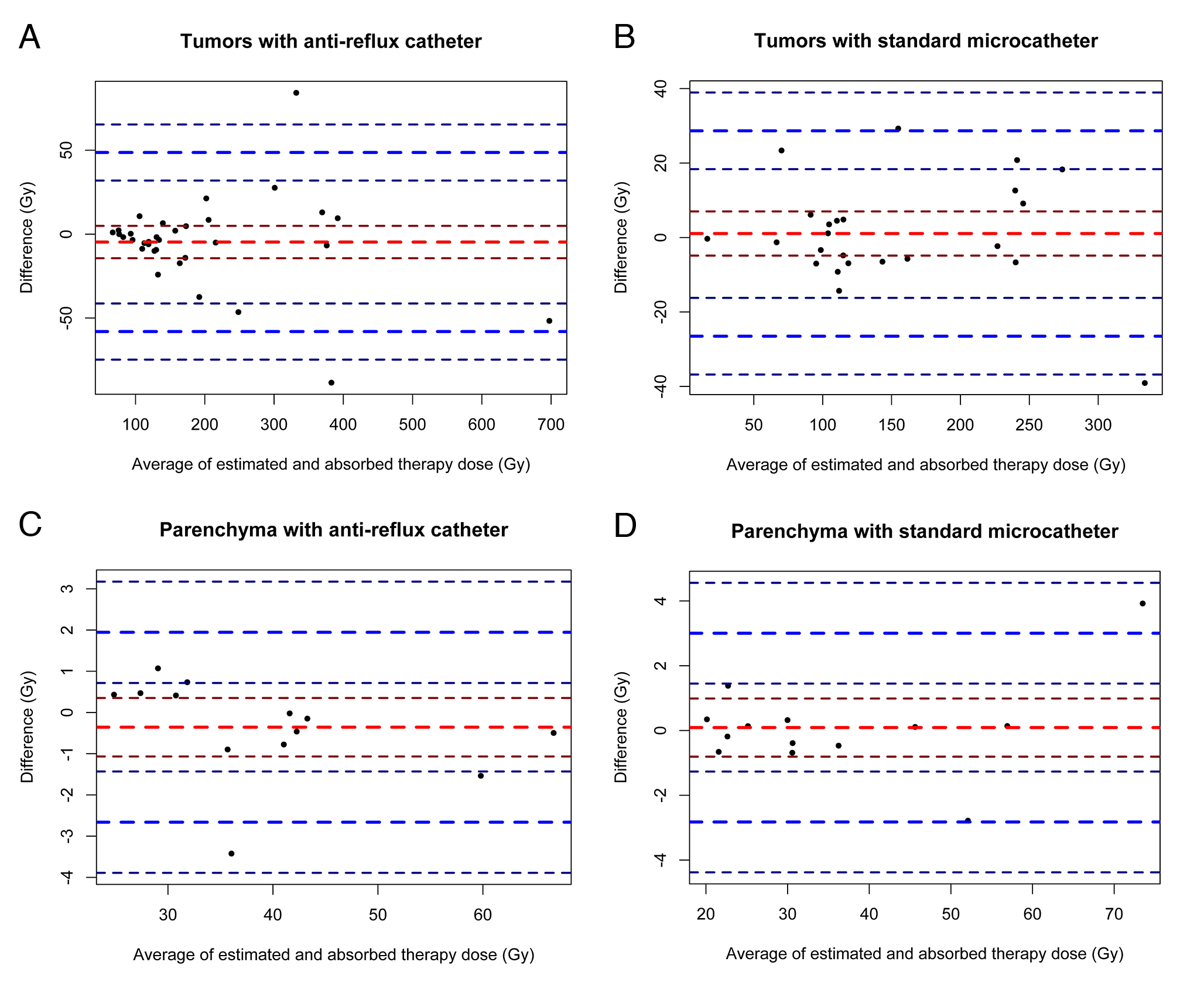

Supplement: Supplementary file 3 — High resolution image (TIF 11041 kb) [file 259_2020_5079_MOESM2_ESM.tif]
