# Supplementary material for: Use of an anti-reflux catheter to improve tumor targeting for holmium-166 radioembolization—a prospective, within-patient randomized study
Source: Eur J Nucl Med Mol Imaging. 2020 Oct 31;48(5):1658–68. doi: 10.1007/s00259-020-05079-0 (PMC8113291; doi:10.1007/s00259-020-05079-0)
Supplement: Supplementary file 4 — (DOCX 13 kb) [file 259_2020_5079_MOESM3_ESM.docx]

| Table S1. Percentage change in mean absorbed dose (Gy) per response category (95%CI) | | | | | |
| --- | --- | --- | --- | --- | --- |
|  | **Progressive disease** | **Stable disease** | **Partial response** | **Complete response** |  |
| *Patient-level* | n=2 | n=11 | n=4 | n=0 |  |
| *Mean dose (Gy)* | *61 (29;129)* | *128 (100;164)* | *150 (102;219)* | - |  |
| Unadjusted | *reference* | 110 0.1;344) | 146 (-14;433) | - | *P_trend_=0.094* |
| *Tumor-level* | n=14 | n=25 | n=8 | n=5 |  |
| *Mean dose (Gy)* | *103 (72;146)* | *121 (94;156)* | *163 (109;243)* | *222 (118;418)* |  |
| Unadjusted | *reference* | 18 (-19;173) | 58 (-4;161) | 116 (8;330) | *P_trend_=0.023* |
| Adjusted*^§^* | *reference* | 21 (-16;80) | 68 (-1;178) | 136 (18;372) | *P_trend_=0.015* |

The dose-response relation was analyzed in seventeen patients that were treated and had availability of both the post-treatment ^166^Ho-SPECT/CT and the three-month follow-up [^18^F]-FDG PET/CT. Interpretation at tumor level: the average dose is 138% higher in CR than PD (95%CI 8;423) (unadjusted analysis).
***^§^***Analysis at a tumor-level was adjusted for catheter type (yes/no anti-reflux catheter)
