# Supplementary material for: Use of an anti-reflux catheter to improve tumor targeting for holmium-166 radioembolization—a prospective, within-patient randomized study
Source: Eur J Nucl Med Mol Imaging. 2020 Oct 31;48(5):1658–68. doi: 10.1007/s00259-020-05079-0 (PMC8113291; doi:10.1007/s00259-020-05079-0)
Supplement: Supplementary file 5 — (DOCX 17 kb) [file 259_2020_5079_MOESM4_ESM.docx]

| Table S2. CTCAE grading of new toxicity per patient during three months from baseline* | | | | | |
| --- | --- | --- | --- | --- | --- |
| Toxicity | **Grade 1** | **Grade 2** | **Grade 3** | **Grade 4** | **Grade 5** |
| *Laboratory toxicity* | | | | | |
| Hypoalbuminemia | **2** | **1** |  |  |  |
| Elevated ALT | **9** | **1** | **1** |  |  |
| Elevated alkaline phosphatase | **2** | **7** | **1** |  |  |
| Elevated AST | **12** | **2** |  |  |  |
| Elevated bilirubin | **1** |  |  | **1** |  |
| Elevated γ-glutamyltransferase | **2** | **8** | **3** |  |  |
| Any laboratory toxicity | **10** | **9** | **3** | **1** |  |
| *Clinical toxicity* | | | | | |
| Abdominal pain | **6** | **9** | **3** |  |  |
| Nausea | **14** | **4** |  |  |  |
| Fatigue | **10** | **8** | **1** |  |  |
| Anorexia | **4** | **1** |  |  |  |
| Dyspnea | **1** |  |  |  |  |
| Fever | **4** | **1** | **1** |  |  |
| Chest pain | **1** |  | **1** |  |  |
| Vomiting | **5** | **2** |  |  |  |
| Back pain | **3** | **1** |  |  |  |
| Bloating | **1** | **1** |  |  |  |
| Joint pain | **4** |  |  |  |  |
| Nocturnal transpiration | **1** |  |  |  |  |
| Diarrhea | **1** |  |  |  |  |
| Constipation | **2** |  |  |  |  |
| Cough |  | **1** |  |  |  |
| Hypotensia |  | **1** |  |  |  |
| Chills | **2** |  |  |  |  |
| Ascites | **1** |  | **2** |  |  |
| Malaise | **3** | **1** |  |  |  |
| Hepatic failure |  |  |  |  | **1**** |
| Weight loss | **5** |  |  |  |  |
| Any clinical toxicity | **6** | **12** | **4** |  | **1** |

CTCAE scores of new laboratory and toxicity during three months after treatment (highest CTCAE grades per laboratory value are represented). *Represented for the 21 patients who underwent treatment. **Radioembolization-induced liver disease.
